# Of Meat and Men: Sex Differences in Implicit and Explicit Attitudes Toward Meat

**DOI:** 10.3389/fpsyg.2018.00559

**Published:** 2018-04-20

**Authors:** Hamish J. Love, Danielle Sulikowski

**Affiliations:** School of Psychology, Charles Sturt University, Bathurst, NSW, Australia

**Keywords:** meat, hunting, implicit attitudes, visual search, masculinity

## Abstract

Modern attitudes to meat in both men and women reflect a strong meat-masculinity association. Sex differences in the relationship between meat and masculinity have not been previously explored. In the current study we used two IATs (implicit association tasks), a visual search task, and a questionnaire to measure implicit and explicit attitudes toward meat in men and women. Men exhibited stronger implicit associations between meat and healthiness than did women, but both sexes associated meat more strongly with ‘healthy’ than ‘unhealthy’ concepts. As ‘healthy’ was operationalized in the current study using terms such as “virile” and “powerful,” this suggests that a meat-strength/power association may mediate the meat-masculinity link readily observed across western cultures. The sex difference was not related to explicit attitudes to meat, nor was it attributable to a variety of other factors, such as a generally more positive disposition toward meat in men than women. Men also exhibited an attention bias toward meats, compared to non-meat foods, while females exhibited more caution when searching for non-meat foods, compared to meat. These biases were not related to implicit attitudes, but did tend to increase with increasing hunger levels. Potential ultimate explanations for these differences, including sex differences in bio-physiological needs and receptivity to social signals are discussed.

## Introduction

Across pre-agricultural hunter-gatherer societies (Cordain et al., 2000; Speth, 2010; Psouni et al., 2012), and through into the agrarian age, (Hayden, 2003) meat may have been the most highly prized food. The expensive tissue hypothesis (Aiello and Wheeler, 1995; Aiello and Wells, 2002) posits that increases in meat and animal fat consumption were central to the evolution of modern humans’ large brain size. Dietary (meat-sourced) fat, beyond the other macronutrients protein and carbohydrate, is important for brain function (Crawford, 1992; Greenwood and Young, 2001), contributes to mental health across the lifespan, and is especially important in early neural development (Cherubini et al., 2007; Innis, 2009; Wainwright, 2002).

In addition to its role in brain development and function, meat consumption may have specific nutritional benefits for adult men. Meat contains creatine, (naturally occurring only in animal-sourced foods), which improves muscular strength, size, and physical and neural performance (Kreider, 2003; Rae et al., 2003; Roitman et al., 2007). Meat has a more complete profile of amino acids than do plant-based proteins (Hoffman and Falvo, 2004). This affords comparatively greater muscle growth and bone density, and thus protection against fractures and other injuries (Bonjour, 2005), to which men are especially prone, due to their greater propensity for risk-taking (Byrnes et al., 1999) and physical violence (Wrangham and Glowacki, 2012; Georgiev et al., 2013). It is also possible that the most nutritionally valuable portions of the kill, such as fat, organ meat and bone marrow, primarily consumed by the hunting males (Berbesque et al., 2011) offset the increased costs and risks of hunting large game (Hawkes et al., 2010).

Meat, sex and gender have been related at least since archaic humans first began increasing consumption of animal source foods, with meat consumption contributing to the structuring of gender roles (Stanford, 1999; Wrangham and Conklin-Brittain, 2003): men generally hunted large game, and women tended to cook it and supplement it with gathered foods. This pattern is also observed across modern hunter-gatherer tribes living in varying ecological contexts where men hunt large high-risk game where available, while women typically gather plant foods and occasionally smaller portions of meat (Bird, 1999). Sex differences and gender roles concerning meat likely have even deeper evolutionary roots. Chimpanzees, like human hunter-gatherers, engage in cooperative hunting and have a resultant social hierarchy, where successful male hunters gain a higher social position and access to meat amongst other males (Boesch, 1994). Larger, more physically robust males typically dominate other males and gain greater status in both human hunter-gatherer tribes and the wider animal kingdom (Hunt et al., 2009). As meat consumption engenders greater physical condition, which in turn may enable better hunting performance, the successful hunting of meat could create a positive feedback cycle, whereby successful hunters may dominate other males in both physical and social status. Successful hunters and providers of meat in human tribes are routinely afforded a greater respect and position by other males in the group, (Wiessner, 1996; Stanford, 1999; Gurven and Hill, 2009).

Strong cross-cultural associations exist between meat and masculinity. Across Europe, Asia, Africa, and the Americas, men consume more meat than do women (Adams, 1990) and across Western societies women are twice as likely as men to be vegan or vegetarian (Trocchia and Janda, 2003; Browarnik, 2012; Ruby, 2012). Furthermore, vegetarianism is seen as a relatively feminine trait (Browarnik, 2012). Meat is typically marketed and modeled as a masculine food (Sobal, 2005; Rogers, 2008) and men with more masculine jobs tend to consume more meat (Roos et al., 2001; Sobal, 2005). Men also self-report more favorable explicit attitudes toward consuming meat (Rothberger, 2013) than do women, while women self-report more disgust and negative attitudes about meat consumption than do men (Kubberød et al., 2002a,b).

Rozin et al. (2012) demonstrated stronger implicit associations between the concepts “meat” and “male” than between “meat” and “female,” and re-affirmed the general link between red meat and masculinity in 4 other implicit and explicit tasks. More importantly, the authors alluded to concepts of strength and power as potentially mediating the link between meat and masculinity, although they did not report data to support this contention, nor did they mention whether the meat-masculinity link differed between male and female respondents. In the first study of the current paper we used an Implicit Association Test (IAT) task to investigate whether meat is associated directly with strength and power, and whether there are sex differences in the strength of this implicit association. In the second study of this paper we employed a visual search task (given the demonstrated links between appetitive motivation and visual attention for food-relevant stimuli, Piech et al., 2010) to investigate whether any sex differences in implicit attitudes observed in the first study could be attributed to concomitant sex differences in appetitive motivation for meat and non-meat foods, respectively.

## Study 1

An Implicit Association Test (IAT) was used to measure strength of associations between meat and health (operationalized using concepts associated with power, strength and vigor) in men and women. An IAT measures reaction times to word/word and/or word/image pairings, with a faster reaction time to a pairing revealing that the participant implicitly connects those two concepts more readily (Greenwald et al., 2003; Lane et al., 2007). The IAT exhibits predictive validity when measuring food attitudes along with eating and buying behavior (Maison et al., 2001; Richetin et al., 2007), and has been shown to measure already formed implicit attitudes as early as age 6, while explicit attitudes continue to change throughout adulthood (Baron and Banaji, 2006). The early formation and stability of implicit associations, as measured by IATs, suggest that they may be less responsive to changing cultural norms than consciously reported attitudes and beliefs. As such, they may be better indicators of predispositions to associate meat with health and vigor, than are explicit, self-reported beliefs about the healthiness of meat. In addition to measuring the association between meat and health (operationalized using terms such as ‘virile,’ ‘strong’ and ‘powerful’), we also measured the association between meat and ’delicious’, in order to ensure that any sex differences observed in the strength of the meat/health association did not simply reflect a general trend for men to more positively evaluate meat than do women, regardless of the specific trait in question. We predicted that, if concepts of power mediate the link between meat and masculinity, as suggested by Rozin et al. (2012), then both men and women would associate meat (more so than non-meat foods) with health. We also set out to explore whether either sex would more strongly associate meat with concepts of power, which Rozin et al. (2012) did not investigate.

### Method

#### Participants

Participants were Australian (primarily) undergraduate students who participated for course credit. Participants completed all tasks described in the current manuscript (beginning with the visual search task, since it was thought least likely to prime specific value judgments that might influence subsequent responses, followed by the IATs and finally, the questionnaire) in a single session, but for ease of presentation, these have been divided into two separate studies. Eighty-three females and 44 males completed the study. Of this sample, the data from 3 females and 1 male was removed from the analysis of Study 1 as these participants made more than 100 errors across the IAT tasks. The final sample, therefore, included 80 females (aged 18–63, *M* = 32.1, *SD* = 10.8) and 43 males (aged 19–60, *M* = 36.4, *SD* = 11.2). Of these, 13 (3 men and 10 women) reported eating no/negligible meat on either the day of participation or the day preceding. All participants gave informed consent under CSU School of Psychology Ethics Committee Approved Protocol No 113/2012/41.

#### Apparatus and Stimuli

The experiment was conducted online, and presented using Inquisit software v3.0.6 and v4.0.0.0, by Millisecond. One IAT assessed the association between images of meat and non-meat foods and the attributes “healthy” and “unhealthy,” and the other assessed the associations between those food types and the attributes “delicious” and “disgusting.” The same 12 meat images (6 fatty meat, and 6 lean meat) and 12 non-meat images (6 vegetables, 6 high carbohydrate foods, including rice, potatoes and breads) were used in both IAT tasks. The attribute word stimuli are presented in **Table 1**.

**Table 1:** IAT attributes stimuli.

IAT 1 attributes		IAT 2 attributes	
Healthy	Unhealthy	Delicious	Disgusting
Energetic	Diseased	Appetizing	Foul
Virile	Drained	Juicy	Rancid
Fit	Weak	Wonderful	Revolting
Strong	Frail	Mouth-watering	Rotten
Vibrant	Feeble	Tasty	Sickening
Powerful	Sick	Yummy	Yucky

#### Procedure

Participants completed the two IAT tasks (healthy-unhealthy and delicious-disgusting) in random order, followed by a series of questions relating to mood, health and diet. During each IAT, participants matched central food images or words with an attribute category listed on the top left (by pressing ‘E’) or top-right (by pressing ‘I’). The healthy/unhealthy IAT began with 48 practice trials. The first 24 of these trials presented participants with a food image (depicting either meat or a non-meat food) in the center of screen and required them to indicate whether the image was meat or non-meat. The words ‘Meat’ and ‘Not meat’ appeared on the top left and right of the screen, respectively, and participants indicated that the image belonged to the ‘Meat’ category by pressing ‘E’ and the ‘Not meat’ category by pressing ‘I’. Accuracy across these practice trials was high with participants averaging 0.7 of an error each, confirming that the images were readily discriminable. The second block of 24 practice trials presented the words ‘Healthy’ and ‘Unhealthy’ on the top left and right of the screen (counter-balanced across participants) and required participants to allocate centrally presented words to either of these categories (the words are presented in **Table 1**).

By the completion of the 48 practice trials, half of the participants had responded with the same key to the meat images and the healthy words, while the other half had responded with the same key to the meat images and the unhealthy words. These respective associations were subsequently tested across two blocks of 48 test trials. During the test trials, all four categories appeared at the top of the screen, two on the left, two on the right, matching the pairings that each participant had experienced in the preceding practice trials. On each trial participants indicated whether a word or picture (the same stimuli presented in the practice trials) belonged to one of the categories on the left (by pressing ‘E’) or one of the categories on the right (by pressing ‘I’). Half of the participants responded with the same key to meat images and healthy words (and non-meat images and unhealthy words), measuring the relative strength of this association, while the remaining participants responded with the same key to meat images and unhealthy words.

Following these test trials the strength of the reverse association was tested for all participants. This commenced with a further 48 more practice trials that were identical to the previous practice trials, except that the category labels ‘Healthy’ and ‘Unhealthy’ now appeared on the opposite sides of the screen. Thus, in the subsequent two blocks of 48 test trials, participants who had previously responded to meat images and healthy words with the same key, were now responding to meat images and unhealthy words with the same key. Word and image trials were interleaved and presented in random order during all blocks of tests trials. If participants responded incorrectly in any practice or test trial, a red cross appeared for 200 ms and participants had to correct their error before continuing. Inter-trial intervals of 250 ms were used (following Greenwald et al., 2003). The delicious/disgusting IAT followed the exact same format as described above, but with the attribute categories ‘healthy’ and ‘unhealthy’ replaced by ‘delicious’ and ‘disgusting.’

D-scores, which indicate the strength and direction of association each participant exhibits between the items (meat and non-meat) and the attributes (healthy and unhealthy, or delicious and disgusting) were calculated for each IAT as follows. Individual trial reaction times less than 300 ms or greater than 10,000 ms were discarded (Greenwald et al., 2003). For trials in which participants responded incorrectly, the self-corrected reaction time was recorded, so that errors were incorporated into the D-score measure via a reaction time penalty. The mean reaction time difference between the first block of test trials and the third block of test trials (the first block after the stimulus-attribute pairing was reversed) was calculated and then divided by the pooled standard deviation of reaction times in across these two blocks. A second D-score was calculated as above for the second and fourth blocks of trials. The average of these two scores was taken as the D-score for that IAT. The sign of D was defined such that positive D-scores indicated faster pairing of ‘meat’ with the attribute ‘healthy’ (and ‘delicious’) and ‘not meat’ with ‘unhealthy’ (and ‘disgusting’) and negative D-scores indicated the opposite patterns. [D-scores were also calculated considering the lean and fatty meat images separately, but the three versions of D-scores correlated very strongly within both males (all *r* > 0.97) and females (all *r* > 0.98), so only the overall meat D-scores were used for subsequent analyses].

#### Surveys

The IATs were followed by a survey in which participants provided their age, sex, height, weight [from which body mass index (BMI) was calculated as weight(kg)/height(m)^2^], hunger levels (on a four-point scale from ‘Not at all hungry’ to ‘Very hungry’), mood (on a six-point scale from ‘Very low’ to ‘Very high’), changeability of mood over the past 2 weeks (on a four-point scale from ‘Changes a lot’ to ‘Hasn’t changed at all’), and listed the food consumed that day and the previous day (via two open-ended questions). Finally, participants were provided with four sets of four images. Each set contained 2 meat images (1 lean and 1 fatty), a carbohydrate image (bread or pasta) and vegetable image (all drawn randomly from those used for the IATs). For two of these sets participants were required to rank the images from least to most healthy and for the other two sets they ranked the images from least to most delicious. The sum of the rank scores (1 = least healthy/delicious, 4 = most healthy/delicious) given to the 2 meat images was calculated and averaged across both image sets (separately for healthy and delicious), such that participants received an explicit rankings score from 2 to 8 for meat healthiness and meat deliciousness.

### Results

All analyses were conducted using SPSS v20 for Mac.

#### Implicit Associations Between Meat, Healthiness and Deliciousness

**FIGURE 1:**
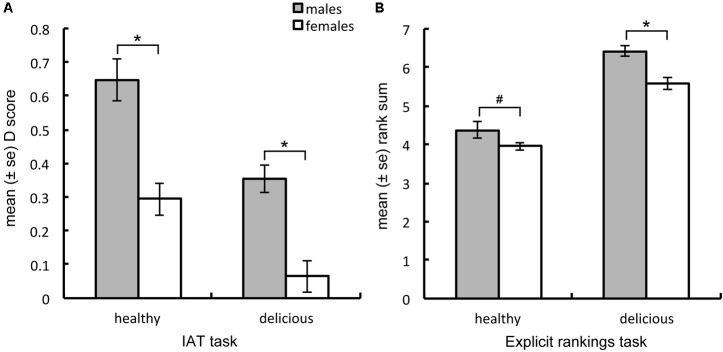
Men exhibited significantly stronger implicit associations **(A)** between meat and the attributes healthy and delicious, respectively, than did women and also tended to explicitly **(B)** rank meat as healthier and more delicious than did women. ^∗^*p* < 0.05, ^#^*p* = 0.067.

**FIGURE 2:**
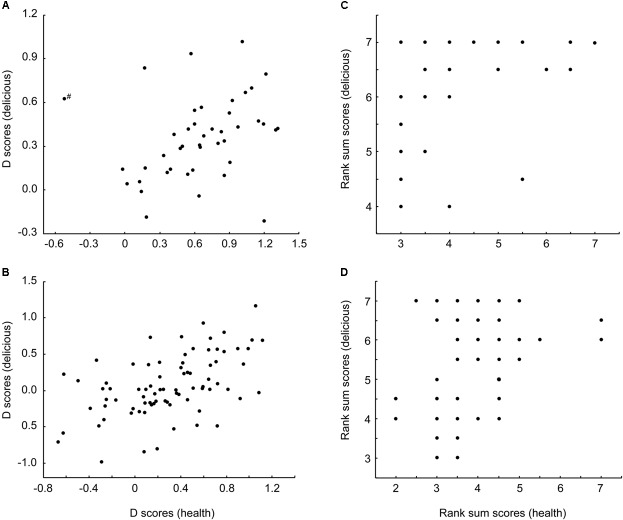
Both implicit **(A,B)** and explicit **(C,D)** measures revealed that both men **(A/C)** and women **(B/D)** exhibited significant positive correlations between the strengths of association between meat and the attributes healthy and delicious, respectively as measured by IAT D scores and explicit rank sums. ^#^Indicates a bivariate outlier that was removed prior to correlation analysis.

One-sample *t*-tests comparing D scores to 0 (which would represent no preferential association between meat and any of the healthy/unhealthy or delicious/disgusting attributes) revealed that both sexes exhibited significant associations between meat and ‘healthy’ [men: *t*(42) = 10.552, *p* < 0.001; women: *t*(79) = 6.108, *p* < 0.001] while only men associated meat with ‘delicious’ [men: *t*(42) = 8.371, *p* < 0.001, women: *t*(79) = 1.376, *p* = 0.173]. Independent-samples *t*-tests confirmed that both of these associations were significantly stronger in men than in women [healthy: *t*(121) = 4.440, *p* < 0.001; delicious: *t*(115.8) = 4.579, *p* < 0.001, adjusted degrees of freedom applied due to violation of non-homogeneity of variance, Levene’s *F*(1,121) = 6.421, *p* = 0.013; see **Figure 1A**]. Both sexes exhibited moderate positive correlations between the ‘healthy’ and ‘delicious’ D scores (women: *r* = 0.519, *n* = 80, *p* < 0.001; men: *r* = 0.399, *n* = 42, *p* = 0.009, one bivariate outlier removed, see **Figures 2A,B**). Since ‘healthy’ and ‘delicious’ D scores were associated (suggesting both could be influenced by a generic positive disposition toward meat), we conducted a univariate ANOVA on the ‘healthy’ D scores with sex as a between subjects factor and ‘delicious’ D score as a covariate. The stronger association between ‘meat’ and ‘health’ in men compared to women, [*F*(1,119) = 10.177, *p* = 0.002], persisted even after controlling for the ‘meat’ and ‘delicious’ association, confirming that the sex difference was not attributable to a general tendency for men to more positively evaluate meat. This same analysis with the bivariate outlier noted above included still revealed a significant main effect of sex [*F*(1,120) = 7.380, *p* = 0.008].

#### Explicit Ratings of Healthiness and Deliciousness of Meat

Independent-samples *t*-tests revealed that men explicitly ranked meat as more delicious than did women [*t*(115.5) = 4.039, *p* < 0.001, adjusted degrees of freedom applied due to non-homogeneity of variance, Levene’s *F*(1,121) = 13.953, *p* < 0.001], but only tended to rank it as healthier than did women [*t*(60.9) = 1.863, *p* = 0.067, adjusted degrees of freedom applied due to non-homogeneity of variance, Levene’s *F*(1,121) = 16.166, *p* < 0.001, see **Figure 1B**]. Both sexes exhibited moderate positive correlations between their explicit meat health and meat deliciousness rankings (men: *r* = 0.321, *n* = 43, *p* = 0.036; women: *r* = 0.406, *n* = 80, *p* < 0.001, see **Figures 2C,D**). One-sample *t*-tests, against a test score of 5 (the rank-sum expected if participants ranked the meat and non-meat images as equally healthy on average) revealed that in contrast to implicit measures both women [*t*(79) = 10.932, *p* < 0.001] and men [*t*(42) = 3.060, *p* = 0.004] ranked the meat as significantly less healthy than the non-meat options, suggesting that the implicit association observed was not solely a reflection of conscious beliefs. Both sexes did, however, rank the meat as significantly more delicious than the non-meat options [women: *t*(79) = 3.783, *p* < 0.001; men: *t*(42) = 10.195, *p* < 0.001].

**FIGURE 3:**
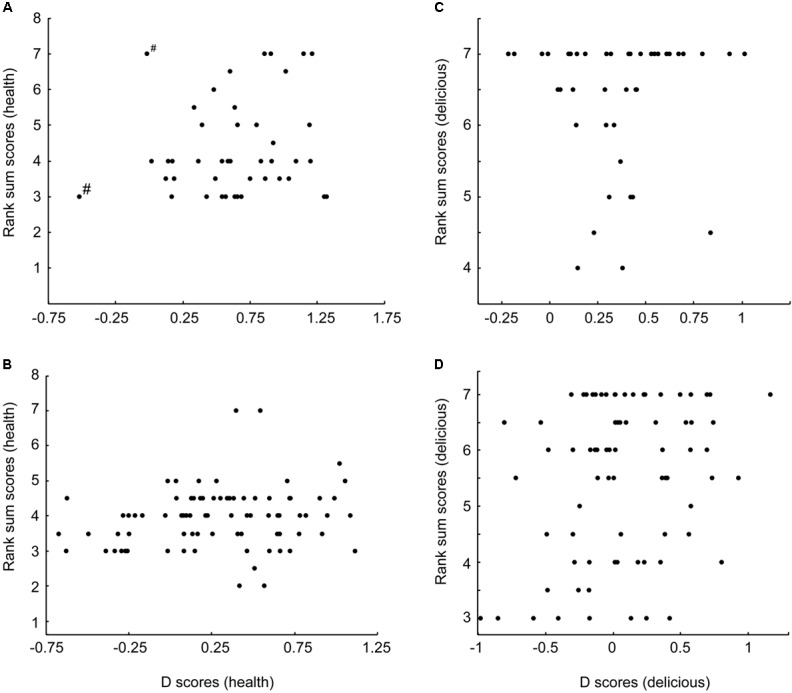
Neither men **(A)** nor women **(B)** exhibited a correlation between the strengths of their implicit and explicit measures of meat healthiness. Women exhibited a positive correlation between their implicit and explicit measures of meat deliciousness **(D)**, while men did not **(C)**. ^#^Removal of these apparent outliers from **(A)** does not reveal a significant correlation, *p* = 0.149.

Correlations were used to directly compare implicit and explicit measures of the healthiness and deliciousness of meat. There was no relationship between implicit and explicit measures of meat healthiness for either sex (men: *r* = 0.174, *n* = 43, *p* = 0.264; women: *r* = 0.184, *n* = 80, *p* = 0.102), confirming that implicit measures of meat healthiness were not simply a reflection of conscious beliefs. Implicit and explicit measures of meat deliciousness were positively correlated for women (*r* = 0.240, *n* = 80, *p* = 0.032), but not for men (*r* = 0.006, *n* = 43, *p* = 0.967, see **Figure 3**).

#### Mood, Hunger and BMI

Neither current mood nor mood changeability scores were correlated with either male or female implicit meat/health or meat/deliciousness association scores (all *r* < |0.182|, all *p* > 0.240). Few hunger scores of 4 (*n* = 4) were reported, so scores of 3 and 4 were combined into a single category and univariate 2 × 3 ANOVAs with hunger level (1, 2, and 3&4) and sex as between-subjects factors were conducted on the meat/health and meat/delicious IAT scores. In neither analysis was the main effect of hunger level or hunger-by-sex interaction significant (all *p* > 0.136). BMI scores were log-transformed to achieve normality. They did not correlate with implicit meat/health associations for women (*r* = 0.116, *n* = 80, *p* = 0.306) or men (*r* = -0.010, *n* = 43, *p* = 0.947), or meat/delicious associations for women (*r* = 0.136, *n* = 80, *p* = 0.229) or men (*r* = -0.059, *n* = 43, *p* = 0.708).

#### Food Consumption

The food consumption reported across both days was scored, such that the scores reflected the relative amount of the diet made up of: lean meats, fatty meats, carbohydrate and vegetables. A score from 1 to 4 was given for each of these food types, with 1 indicating absence/negligible presence of that food type, 2 indicating the food type was present in low quantities, 3 indicated the food type was repeatedly eaten that day, and 4 indicated the food type was the dominant component of the diet. Author HL applied the scores, without knowledge of participants’ D-scores (which were calculated independently by author DS). Scores from both days were summed such that each participant received a score for each food type that ranged from 2 to 8. We then averaged the two meat scores and two non-meat scores. One male participant failed to provide his food consumption, so only 42 males are included in the following analyses.

An independent-samples *t*-test confirmed that men ate significantly more meat (proportionately, compared to non-meat) than did women [*t*(61.523) = 3.054, *p* = 0.008; adjusted degrees of freedom applied due to unequal sample variances, Levene’s *F*(1,120) = 8.773, *p* = 0.004]. Correlation analyses revealed that the amount of meat eaten was positively associated with explicit ratings of meat healthiness in both women (*r* = 0.253, *n* = 80, *p* = 0.024) and men (*r* = 0.639, *n* = 42, *p* < 0.001), with this association significantly stronger in men (*z* = 2.533, *p* = 0.011); and with explicit ratings of meat deliciousness in women (*r* = 0.291, *n* = 80, *p* = 0.009), but not in men (*r* = 0.229, *n* = 42, *p* = 0.144), with no sex difference in the strength of these associations (*z* = 0.334, *p* = 0.738), suggesting a lack of power might be responsible for the male null result. There were no significant correlations between the amount of meat eaten and implicit meat/health associations for either women (*r* = 0.071, *n* = 80, *p* = 0.532) or men (*r* = 0.063, *n* = 42, *p* = 0.693), but amount of meat eaten did tend to correlate with implicit meat/deliciousness associations for both women (*r* = 0.218, *n* = 80, *p* = 0.052) and men (*r* = 0.301, *n* = 42, *p* = 0.052). The positive association between meat eaten and implicit meat/deliciousness associations was significant when data from both sexes was combined (*r* = 0.301, *n* = 122, *p* = 0.001).

### Discussion

The IAT demonstrated that both men and women associated ‘meat’ and ‘healthy’ more strongly than ‘non-meat’ and ‘healthy,’ suggesting that concepts of strength and power could mediate the meat-masculinity associations that have been reported elsewhere (Rozin et al., 2012). There was also a significant sex difference, with men exhibiting a stronger implicit link between ‘meat’ and ‘healthy’ than do women. This sex difference persisted when associations between ‘meat’ and ‘delicious’ were controlled for, confirming that it did not arise out of a generic tendency of men to evaluate meat more positively than do women. Further, the strength of the meat-healthy association did not correlate with explicit judgments about the healthiness of meat, confirming that it does not simply reflect sex differences in how healthy the participants consciously believe meat to be. These associations also did not correlate with the amount of meat that participants reported they had eaten over the last 2 days, suggesting that sex differences in the amount of meat consumed, or in the propensity to be vegetarian, are also not likely to be responsible for the greater male propensity to associate ‘meat’ with ‘healthy.’ Lastly, this sex difference was also not attributable to sex differences in mood, hunger or BMI, as all three of these measures were unrelated to implicit meat/health associations.

Although men tended to rate meat as both more delicious and healthier than did women, in both implicit and explicit measures, there were no significant associations between implicit and explicit measures of the healthiness of meat, for either men or women, and only women exhibited a significant correlation between implicit and explicit measures of meat deliciousness. Why women’s implicit and explicit attitudes about the taste of meat would correlate, but men’s would not, is not immediately clear. Implicit measures of meat deliciousness positively predicted how much meat both men and women had consumed over the last 2 days. That men’s implicit attitudes about the taste of meat better predict their eating behavior than their explicit attitudes could reflect that men have incorporated cultural norms about the masculinity of meat into their conscious attitudes about their taste preferences for it. This may have inflated the explicit deliciousness scores (whose mean was close to the maximum score) obscuring any correlation via a ceiling effect. If this is the case, it is consistent with suggestions made by other authors that implicit attitudes are less impervious to changing cultural norms and learnt beliefs than are explicit attitudes (Baron and Banaji, 2006).

Strong associations between ‘meat’ and ‘healthy’ in men could reflect their own functional motivations for hunting and consuming meat. Given the physical benefits of meat consumption in terms of musculature and bone density (Bonjour, 2005), and the importance of physical stature in male-male social status competition (Manson and Wrangham, 1991), implicit associations between ‘meat’ and ‘healthy’ may form part of the motivation that lead men to consume more meat than women. In Study 2, we explored whether sex differences in implicit appetitive motivations for meat could account for the sex differences in the strengths of implicit associations between ‘meat’ and ‘healthy.’

## Study 2

We have already discussed the greater physiological benefits to men, relative women, of eating meat, and there may also be specific physiological benefits to women of eating some non-meat foods. Despite a modern societal prejudice against fat on female bodies (Harris and Smith, 1983; Turnbull et al., 2000), bodily fat is vitally important for female health, including reproductive health (Troisi et al., 1995; Lassek and Gaulin, 2007; Jayasinghe et al., 2008) and the developmental health of a mother’s child (Innis, 2000, 2007; Uauy and Hoffman, 2000). The tendency to store and hold fat in easily metabolisable gluteal stores (on the thighs and buttocks) may facilitate a mother’s ability to express highly nutritious milk for children (Power and Schulkin, 2008). Perhaps surprisingly, there is little evidence that direct animal fat consumption contributes effectively to female fat stores, with growing evidence suggesting that a high carbohydrate diet is most effective in stimulating the body to store excess energy as fat (Ludwig et al., 2001; Taubes, 2001; Samaha et al., 2003; Schulze et al., 2004; Johnson et al., 2007; Mente et al., 2009). This would tend to suggest that meat consumption is unlikely to be of unique benefit to women, but may explain greater female, relative to male, craving of carbohydrate-based foods (Grogan et al., 1997).

Stimuli of high relevance capture attention faster than stimuli of lower relevance in a visual display (Brosch et al., 2008). Threat-relevant stimuli, such as snakes and spiders (Öhman et al., 2001; LoBue, 2010; Sulikowski, 2012), and guns and knives (Brosch and Sharma, 2005; Sulikowski and Burke, 2014), are routinely found more quickly than similar non-threatening stimuli in visual search tasks. Baby faces, as a highly relevant positive stimulus, also preferentially attract attention (Brosch et al., 2007). We adopted a visual search task as visual attention toward food stimuli has repeatedly been used as an indicator of appetitive motivation. Food-deprived people exhibit preferential visual attention for food stimuli, as measured by increased gaze duration (Castellanos et al., 2009), event-related brain potentials (Stockburger et al., 2009b) and during an attentional blink paradigm (Piech et al., 2010). Vegetarians exhibit a stronger neural response, indicative of visual attention, to meat images, specifically, than do non-vegetarians (Stockburger et al., 2009a). The authors interpreted this effect as indicating a stronger aversive emotional response to meat in vegetarians compared to omnivores, but the finding is equally consistent with the notion that individuals requiring more animal source fats and proteins in their diet preferentially orient toward such foods, assuming that vegetarians are more likely than omnivores to be deficient on such nutrients.

More recently, visual search paradigms have been modified to permit a measurement of caution, as well as speed, while responding (Sulikowski, 2012; Sulikowski and Burke, 2014). This caution score reflects the extent to which participants delay their response during trials that do not contain a target: the longer a participant waits before indicating that a target is indeed absent (relative to the average time it takes them to locate the target when it is present), the higher is the cost the participant has implicitly placed on missing such a target. Using this method, higher levels of caution have been observed during visual search for potentially lethal (compared to non-deadly) spiders (Sulikowski, 2012). Higher levels of caution have also been observed when searching for weapons (guns and knives), compared to non-weapon objects, with further increases in caution observed if the weapons are depicted wielded (Sulikowski and Burke, 2014). In the present study we administered a visual search task in which participants searched for both meat and non-meat food images. We measured response time to locate these targets, typically presumed to indicate the immediate relevance of the target to participants (as it is affected by current motivational state as well as contextual factors, Neider and Zelinsky, 2006), as well as the levels of caution expressed during search as an implicit measure of the value participants assign to the different food types (Sulikowski, 2012).

Considering the sex-specific benefits of meat and non-meat consumption, and previous studies linking visual attention to appetite, if meat is a more valuable source of nutrition for men than for women, then we predict men to locate meat images faster and with more caution compared to non-meat images, with the reverse pattern appearing for women. We would further predict increased hunger to then have a stronger influence on male response times and caution exhibited when locating meat (speeding up response times and increasing caution), compared to non-meat images, with the reverse pattern appearing for women. Critically, if the sex differences in implicit attitudes about the healthiness and deliciousness of meat observed in Study 1 are due to sex differences in appetitive motives, we would predict correlations between the D scores observed in Study 1, on the one hand, and the reaction time and caution levels exhibited when locating the meat images in Study 2, on the other.

### Method

#### Participants

Of the 83 females and 44 males that completed the task, data from 8 females and 1 male were not included in the analysis of Study 2 as they returned an accuracy score of zero in at least one condition of the visual search task. The sample for Study 2, therefore comprised the same 43 males from Study 1 and 75 (aged 18–62, *M* = 31.6, *SD* = 10.2) of the 80 females from Study 1.

#### Stimuli

The visual search task contained four conditions defined by the type of food being searched for: fatty meats, lean meats, vegetables and high carbohydrate foods (which included rice, pastas, potatoes and breads). Nine target images, a unique image for each trial, (10 × 7 cm and presented at a resolution of 72 dpi) were used in each target category. These 9 images included the 6 images used for each these categories during the IAT tasks of Study 1. The distracter images used were drawn from nine categories – clocks, shells, plants, books, rocks, flowers, shoes, cats, and bowls. Nine different images from each distractor category were also used across the task.

#### Procedure

The visual search task comprised four blocks of 18 trials. A different target type (fatty meats, lean meats, high carbohydrate foods, and vegetables) was defined for each of the four blocks, and within the each block there were 9 target-present and 9 target-absent trials. The order of the blocks, and the trials within the blocks, was randomized for each participant.

Each trial presented participants with a fixation cross (for 700 ms), followed by a 3 × 3 grid of 9 images (either 9 distracter images from 9 different categories, or 8 distracters from 8 different categories plus one target). Participants had to respond as quickly as they could to indicate if the nominated target was present (pressing the ‘p’ key) or absent (pressing ‘a’ key). The images remained on the screen until participants responded at which point a 400 ms inter-trial interval occurred followed by the fixation cross for the subsequent trial. In each of the four blocks, the target appeared in each of the 9 possible locations exactly once, the placement of distractor images was randomized.

Reaction time for each of the four blocks was calculated as the mean response time for target present trials for which the participant provided a correct response, with individual trial response times shorter than 250 ms and longer than 5000 ms excluded. These criteria excluded three individual response times (across two participants) for being too short, and 142 response times (across 47 participants) for being too long (a total of 1.7% of response times removed across all participants). Mean accuracy across all conditions was very high (93.8–99.4% for absent trials, and 85.3–92.2% for present trials), within individual participant accuracy across the task ranging from 81 to 100%. Following Sulikowski (2012), caution was calculated as (A-P)/(A+P), where ‘A’ is the mean correct response time for target-absent trials and ‘P’ is the mean correct response time for target-present trials. This creates a standardized score that reflects the proportionate increase in response time from target-present to target-absent trials, reflecting how long a participant delays their ‘absent’ response, to reduce the probability of a ‘miss’ error. The higher the caution score, the longer the participant is waiting (beyond the time it typically takes them to locate the target), to declare it absent, and thus the more cost they are implicitly placing on missing a potentially present target.

### Results

#### Effects of Sex and Hunger Levels

**FIGURE 4:**
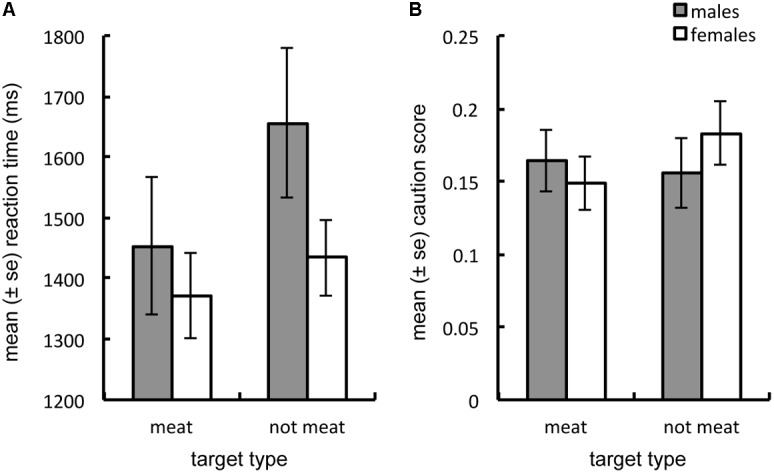
Averaged across hunger levels, men exhibited a larger response time advantage to locate meat over non-meat, than did women **(A)**. Men also exhibited greater caution toward meat, while women exhibited greater caution toward non-meat **(B)**. Both univariate food-type by sex interactions were significant, *p* < 0.05.

We conducted a mixed-effects MANOVA on mean reaction times and caution scores when locating the meat and non-meat targets, with target-type (2 levels: meat and non-meat) as a within-subjects variable and sex and hunger level as between-subjects variables (with hunger level defined as described for Study 1). As predicted, we observed a significant sex-by-target type interaction [*F*(1,111) = 3.531, λ = 0.940, *p* = 0.033], which univariate tests confirmed was significant for both response times [*F*(1,112) = 5.975, *p* = 0.016] and caution scores [*F*(1,112) = 4.769, *p* = 0.031] considered separately. These interactions occurred as men responded more quickly to meat compared to non-meat (*p* < 0.001), while women did not (*p* = 0.089), and women responded more cautiously to non-meat than to meat (*p* = 0.006), while men did not (*p* = 0.578, see **Figure 4**).

**FIGURE 5:**
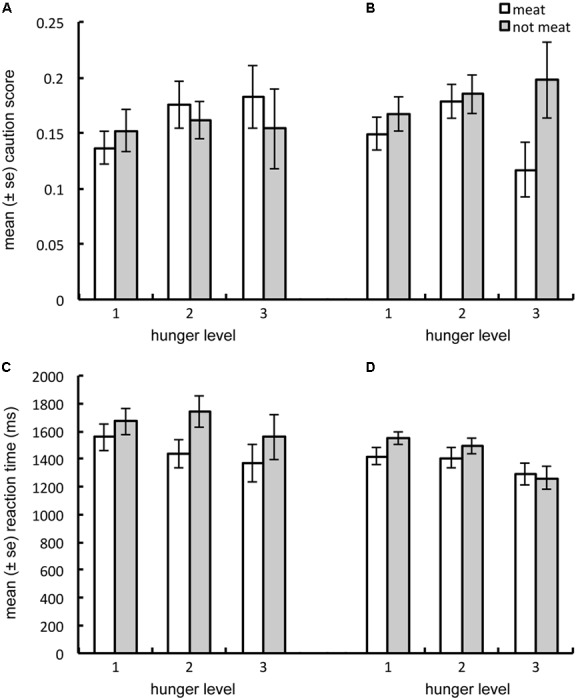
Shows that as hunger increases, men tend to increase caution **(A)** and decrease reaction time **(C)** in response to meat, compared to non-meat; while women to increase caution **(B)** and decrease reaction time **(D)** to non-meat, compared to meat images. The multivariate three-way interaction between hunger level, sex and food-type, approached significance only, however, *p* = 0.061.

To determine whether state hunger was a stronger driver of visual attention for meat in men, compared to women, we examined the three-way sex-by-hunger-by-target type interaction. This interaction term trended in the predicted direction [*F*(1,222) = 2.262, λ = 0.923, *p* = 0.061], as males exhibited a larger decrease in response times and increase in caution scores for meat compared to non-meat images as hunger levels increased. For females, these same changes were larger for non-meat than for meat images (see **Figure 5**).

#### Correlations Between Reaction Time, Caution and Implicit Associations

To determine whether participants’ response times or the levels of caution expressed when searching for meat and non-meat foods, respectively, predicted their implicit attitudes (which would imply that the implicit attitudes may be driven by appetitive motives) correlations between response times, caution scores and implicit meat/health and meat/deliciousness associations (D scores from Study 1) were examined. For women there were no significant correlations between either response times or caution scores (when searching for meat or non-meat food images) and either the ‘healthy’ or ‘delicious’ D scores (all *r* < |0.084|, all *p* > 0.481, *n* = 72). For men, no significant correlations emerged between response times (when searching for either meat or non-meat images) and either ‘healthy’ or ‘delicious’ D scores (all *r* < |0.111|, all *p* > 0.481, *n* = 42). Caution scores when searching for meat images (*r* = -0.313, *p* = 0.043, *n* = 42) and non-meat images (*r* = -0.328, *p* = 0.034, *n* = 42) were negatively correlated with ‘healthy’ D scores, indicating that male participants with a stronger implicit association between meat and health tended to exhibit less caution when searching for all food types in the visual search task (no such relationships appeared between the caution scores and meat/delicious associations, both *r* < |0.189|, both *p* > 0.234).

### Discussion

The sex-by-food type interaction, wherein men responded more quickly to meat than non-meat images, and women responded more cautiously to non-meat than to meat images, suggests that men implicitly evaluated the meat images as more immediately relevant than the non-meat images, and that women placed a higher cost on missing the non-meat, than the meat, images. It is important to note thought that response time, (though not caution, Sulikowski, 2012) in visual search tasks is susceptible to low-level visual confounds between stimuli categories, (Quinlan, 2013). In the current study both men and women located the meat images more quickly than the non-meat images (only men, significantly so). It is possible that the meat images were simply easier to perceive amongst the distractors than the non-meat images. So while the sex difference implies that men do indeed direct visual attention to meat images more so than to non-meat images, relative to women, the null result observed for women, could indicate the absence of an attentional bias, or it could indicate a bias for non-meat foods, that has been offset, by the greater visual salience of the meat images.

The patterns in response and time and caution observed are consistent with the proposed sex differences in relative appetitive motivations for meat and non-meat, as appetitive motivations have previously been linked with biases of visual attention toward food (Castellanos et al., 2009; Stockburger et al., 2009b; Piech et al., 2010). That these sex differences were further exacerbated, albeit equivocally, by state hunger, also suggests a direct link between nutritional needs, appetitive motivations and sex differences in the psychology of responses to meat.

Conversely, we found no evidence to suggest that the sex differences in implicit attitudes reported in Study 1, were the result of sex differences in relative appetitive motivations for meat and non-meat foods. The implicit measures of association between meat and health (D-scores) did not vary as a function of self-reported hunger (results of Study 1). They were also unrelated to the mean response times to locate meat images, for either sex in Study 2. Although there were significant correlations between the implicit meat – health association scores from Study 1 and the caution scores from Study 2 (for men only, not for women), this does not imply a direct relationship between appetitive motivation and implicit attitudes for two reasons. The correlations occurred between the meat – health implicit associations and between the caution scores for both meat and non-meat foods. An appetitive explanation would predict a relationship only between the caution scores when searching for meat, not when searching for non-meat images.

Secondly, and most importantly, the direction of the correlations is in the opposite direction to that predicted by an appetitive motivation account. Men exhibited less caution when searching for the meat and non-meat images, the stronger was their implicit association between meat and health. This is in spite of men exhibiting relatively more caution when searching for meat than non-meat images, compared to women, and exhibiting stronger appetitive motivations for meat generally across both studies (as indicated by their relatively faster responses to meat images in the visual search task, greater self-reported consumption of meat and the sex differences in implicit and explicit attitudes toward meat deliciousness). The negative correlations between ‘healthy’ D scores and caution during visual search, therefore, require some other explanation.

One possibility is that both measures are affected by individual differences in the overall masculinity, or intrasexual competitiveness, of male participants. The caution score, in effect, measures how long a participant waits during target absent trials, before responding that they are sure that the target is indeed absent. Within-participant differences in this measure in response to different categories of targets can be interpreted as differences in an implicit judgment of how costly it would be to miss a target (Sulikowski, 2012; Sulikowski and Burke, 2014). There are, however, also quite large individual differences in this measure and these could indicate a participant’s general risk-proneness or risk-aversion, with more risk-prone participants responding more quickly in target-absent trials, generally. Men are typically more risk-prone than women (Byrnes et al., 1999) and risk-proneness in men is predicted by 2D:4D ratio – a marker of prenatal testosterone exposure (Stenstrom et al., 2011), that is also associated with physical aggression in men (Bailey and Hurd, 2005). A recent study also links risk-proneness in males, with greater perceived competitive formidability (Fessler et al., 2014). This suggests that more masculine, more intra-sexually competitive men, may also be more risk-prone. The strength of implicit associations between ‘meat’ and ‘healthy’ in men could also reflect engagement in intrasexual competitiveness (similar to how men with more masculine jobs also eat more meat, Roos et al., 2001; Sobal, 2005). Thus, the correlations between caution scores and ‘healthy’ D scores could derive from both measures reflecting individual differences in male competitiveness. Further investigations directly comparing these measures with measures of individual masculinity need to probe this possibility before firm conclusions can be drawn.

## General Discussion

In Study 1, we observed strong implicit associations between ‘meat’ and ‘healthy’ in both sexes, suggesting that a meat-power/strength link may mediate previously reported meat-masculinity associations. We also observed, however, strong sex differences, favoring men, in the strength of this association. The results of Study 1 and Study 2 combined rule out a number of sexually dimorphic psychological and behavioral factors related to meat, and to food consumption more generally, as likely drivers of the sex difference in the meat-health association. These include meat consumption, BMI, mood, hunger and explicit knowledge and beliefs about the healthiness of meat. With respect to the visual search task applied in Study 2, there are good reasons to suspect that the sex differences observed in caution and response time reflect sex differences in appetitive motivations. The opposing food-type specific effects observed in each sex, tended to increase as participants’ self-reported hunger levels increased and several previous studies (Castellanos et al., 2009; Stockburger et al., 2009b; Piech et al., 2010) have linked visual attention to appetitive motivations. There were, however, no relationships between implicit association strengths and either visual search response times or self-reported hunger levels.

Since the sex differences in the ‘meat’ and ‘healthy’ associations observed in Study 1 are not easily attributable to sex differences in appetitive motivations for meat and not accounted for by the various other measures included in the current study, then another explanation for these differences is required. One possibility relates to the role of male meat provisioning as a potential social signal. A man’s hunting prowess may serve as a signal to others in the group (Hawkes and Bliege Bird, 2002). Within the Ache (Hill and Hurtado, 1996), Hadza (Marlowe, 1999), !Kung (Wiessner, 2002), people of Lamalera, Indonesia (Alvard and Gillespie, 2004) and the Meriam of the Torres Strait (Smith et al., 2003) hunting success in males has been linked with higher fertility, younger wives, more sexual partners and/or lower child mortality (reviewed by Smith, 2004) and women from the Hadza tribe listed hunting skill as the single most desired trait in a partner (Marlowe, 2004). As such women and other men may perceive successful hunters as attractive potential mates or fierce mating rivals, respectively.

Receiver psychology (Rowe, 1999) refers to the psychological adaptations of individuals that permit them to effectively perceive (receive) social signals. In the case of hunting, relatively few individuals would directly witness a successful hunt, but many would witness the spoils. It may therefore be the case that meat provisioning, rather than hunting itself, constitutes the signal of male prowess. Implicit associations between the ‘meat’ and ‘healthy’ might reflect aspects of receiver psychology that automatically link meat, and by extension, the men who provide it, with concepts of strength and vigor. If this is the case, it could explain why both men and women implicitly associated ‘meat’ more so than ‘non-meat’ with ‘healthy’ (in spite of women explicitly reporting the meat to be less healthy than the non-meat, consistent with previous reports, Beardsworth et al., 2002). Given the stronger meat-healthy associations exhibited by men, it could also be the case that men are more sensitive to the signaling qualities of meat than are women.

Further evidence that these implicit measures linking meat with ‘health’ reflect sensitivity to meat’s signaling capacity in this way, could be obtained by comparing them with individual differences in mating motivations. For example, changes in these implicit association strengths that correlate with changes in masculinity or socio-sexual orientation in men, or with cycling fertility changes or pregnancy in women, would be especially compelling.

A further possibility is that the strong association between meat and ‘health’ displayed by men, reflects psychological motivations to provision. Future studies could compare the strength of the meat-health association displayed by men at different life phases, where intra-sexual competition and provisioning for children are differentially important. Evidence for this possibility would be obtained if the association strengthens, rather than weakens, as men age and have more children.

Consistent with previous studies, men reported eating proportionately more meat than did women (Adams, 1990) and explicit attitudes toward meat were more favorable for men than for women (Kubberød et al., 2002a,b). Explicit attitudes about the healthiness of meat also predicted actual dietary behavior, consistent with the findings of Rothberger (2013), who also observed that males were more likely to justify consumption of meat for health reasons than were females. Interestingly, implicit, but not explicit, associations between meat and deliciousness also tended to predict meat consumption behavior, for both sexes. This suggests that meat consumption may be closely associated with biological taste preferences (as revealed by implicit measures) and that explicit attitudes may exist as justifications for meat consumption. This interpretation is consistent with the findings and conclusions of Rothberger (2013) who examined the justifications offered by males and females for consuming meat (in spite of the animal welfare concerns). He reported that more masculine men more strongly endorsed direct justifications for eating meat, such as enjoying eating it, and also reported eating more meat.

### Summary and Conclusion

In the present study we observed a series of sex differences in implicit responses to meat and non-meat foods. Men exhibited stronger associations than did women between meat and the concepts ‘healthy’ and ‘delicious,’ prioritized visual attention for meat more so than did women, and searched for images of meat more cautiously than did women, suggesting that they implicitly value meat more highly than do women. The data support suggestions from Rozin et al. (2012), that concepts of power and strength might mediate the meat-masculinity link they observed. To the extent that hunting and meat provisioning can act as a social signal our data suggest that the primary targets of that signal are other men, with women acting potentially only as secondary receivers. It is also possible, however, that sex-differences in the physiological benefits of meat consumption drive the sex differences in implicit responding that we report here, independently of any social-signaling value that hunting and meat provisioning may hold. Further comparisons of these types of implicit measures between groups of participants that differ in their state physiological requirements for meat, and their supposed receptivity to social signals of hunting and meat provisioning, are needed to illuminate these possibilities.

## Ethics Statement

This study was carried out in accordance with the recommendations of the National Statement on Ethical Conduct in Human Research, The (Australian) National Health and Medical Research Council with informed consent from all subjects. All subjects indicated informed consent online, rather than in writing, as the study was completed online. The protocol was approved by the Charles Sturt University, School of Psychology Ethics Review Committee.

## Author Contributions

HL designed and conducted the study and contributed to analysis. DS contributed to design of the study, assisted in data collected, and conducted the analysis. The authors contributed equally to the written manuscript.

## Conflict of Interest Statement

The authors declare that the research was conducted in the absence of any commercial or financial relationships that could be construed as a potential conflict of interest.
